# Can spatial self-organization inhibit evolutionary adaptation?

**DOI:** 10.1098/rsif.2024.0454

**Published:** 2025-01-29

**Authors:** B. K. Bera, O. Tzuk, J. J. R. Bennett, U. Dieckmann, E. Meron

**Affiliations:** ^1^ The Swiss Institute for Dryland Environmental and Energy Research, BIDR, Ben-Gurion University of the Negev, Midreshet Ben-Gurion 8499000, Israel; ^2^ Physics Department, Ben-Gurion University of the Negev, Beer-Sheva 8410501, Israel; ^3^ Icahn School of Medicine at Mount Sinai, New York, NY 10029, USA; ^4^ Complexity Science and Evolution Unit, Okinawa Institute of Science and Technology Graduate University (OIST), Onna 240-0495, Japan; ^5^ Advancing Systems Analysis Program, International Institute for Applied Systems Analysis (IIASA), Laxenburg A-2361, Austria; ^6^ Research Center for Integrative Evolutionary Science, The Graduate University for Advanced Studies (Sokendai), Hayama 240-0193, Japan

**Keywords:** drylands, vegetation pattern formation, evolutionary adaptation, mathematical modelling, homeostasis, trade-off

## Abstract

Plants often respond to drier climates by slow evolutionary adaptations from fast-growing to stress-tolerant species. These evolutionary adaptations increase the plants’ resilience to droughts but involve productivity losses that bear on agriculture and food security. Plants also respond by spatial self-organization, through fast vegetation patterning involving differential plant mortality and increased water availability to the surviving plants. The manners in which these two response forms intermingle and affect productivity and resilience have not been studied. Here we ask: can spatial patterning inhibit undesired evolutionary adaptation without compromising ecosystem resilience? To address this question, we integrate adaptive dynamics and vegetation pattern-formation theories and show that vegetation patterning can inhibit evolutionary adaptations to less productive, more stress-tolerant species over a wide precipitation range while increasing their resilience to water stress. This evolutionary homeostasis results from the high spatial plasticity of vegetation patterns, associated with patch thinning and patch dilution, which maintains steady local water availability despite decreasing precipitation. Spatial heterogeneity expedites the onset of vegetation patterning and induces evolutionary homeostasis at an earlier stage of evolutionary adaptation, thereby mitigating the productivity loss that occurs while the vegetation remains spatially uniform. We conclude by discussing our results in a broader context of evolutionary retardation.

## 1. Introduction

The response of ecosystems to climate change is likely to involve ecological processes occurring at different organizational levels, trophic levels and time scales [1–6]. One process of this kind that receives increasing attention is spatial self-organization in regular and irregular vegetation patterns [7–9], partly because of its substantial role in increasing ecosystem resilience to environmental stressors such as droughts [10–12]. Vegetation pattern formation is a population-level process involving differential plant mortality, which increases resource availability to the remaining plants. However, the manners by which the collective dynamics of vegetation patterning intermingle with other processes in response to environmental stressors, including phenotypic changes at the individual plant level, community reassembly or evolutionary adaptation, have received little attention despite their likely occurrence [13–20].

In this paper, we study the coupled responses of dryland vegetation to drying climates involving slow evolutionary adaptation and fast vegetation patterning. As vegetation pattern formation is a threshold phenomenon, occurring when the precipitation drops below a threshold value, we ask how the slow evolutionary adaptation of spatially uniform vegetation to water stress changes once the threshold is traversed and spatial patterns appear. Adaptation to water stress can occur in various ways [21], including reduction in leaf area to reduce water loss, root extension to reach moister soil layers, changes in photosynthetic pathways, e.g. from C3 plants to crassulacean acid metabolism (CAM) plants and others. These processes not only reduce plant mortality but also slow down plant growth because of reduced CO_2_ assimilation and photosynthesis, higher resource allocation to roots, temporal separation of CO_2_ assimilation and Rubisco activity, etc. Thus, a trade-off between plant growth and tolerance to water stress generally exists [22–24]. This trade-off suggests the possibility of slow evolutionary adaptation from fast-growing to stress-tolerant species as water stress develops. Such evolutionary adaptation increases the resilience of ecosystems but reduces their productivity, thus bearing on agriculture and food security and raising the question: can spatial patterning inhibit undesired evolutionary adaptation without compromising ecosystem resilience?

**Figure 1.**
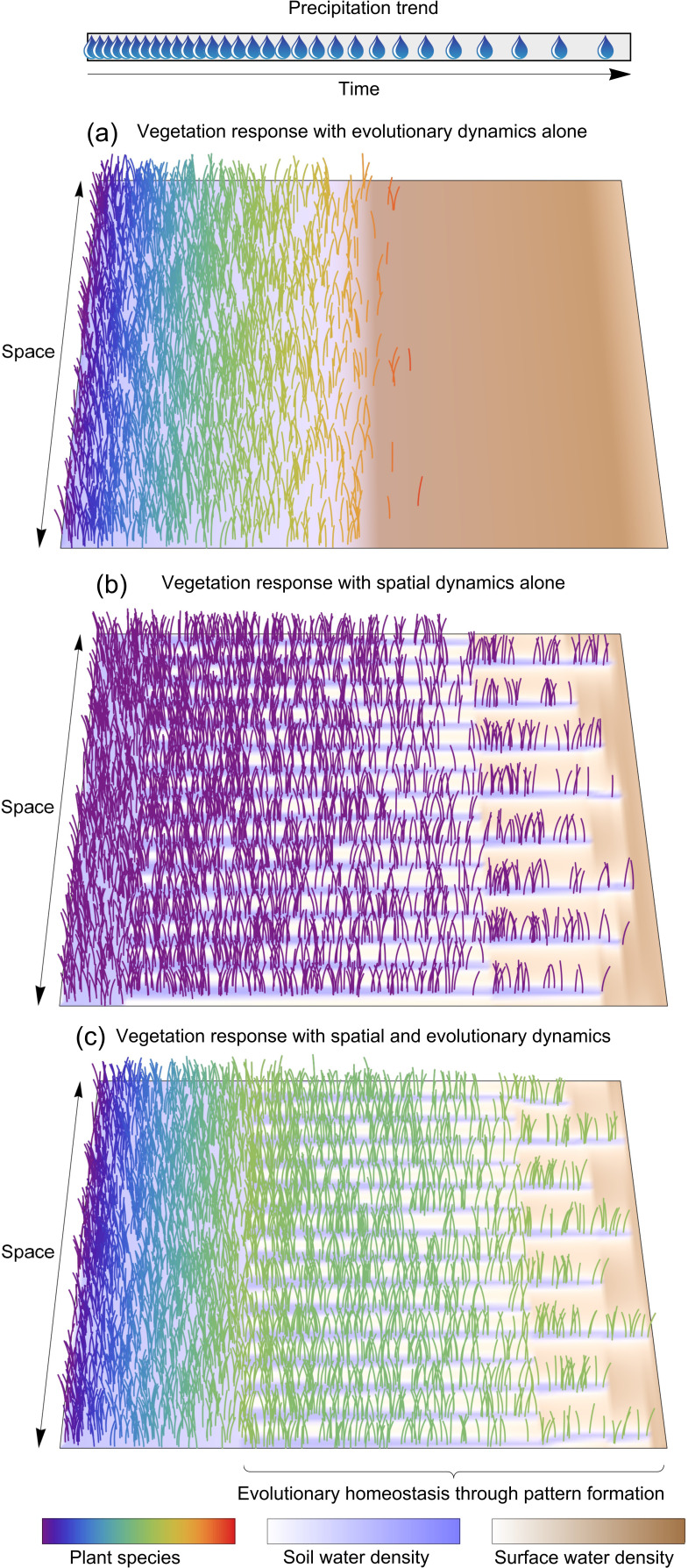
Vegetation responses to decreasing precipitation. (*a*) When the response of a fast-growing species (bluish colours) involves evolutionary adaptation alone, a gradual evolution making the species more tolerant to water stress (reddish colours) occurs until the ecosystem collapses to bare soil. (*b*) When the response of a fast-growing species involves spatial patterning alone, an initial state of uniform vegetation survives the increasing water stress by forming a periodic pattern followed by transitions to longer-wavelength patterns. That response results in improved resilience, as the collapse to bare soil occurs at a significantly lower precipitation threshold than for evolutionary adaptation alone. (*c*) When both mechanisms act together, the ecosystem’s resilience to decreasing precipitation is yet improved, but, more importantly, the evolutionary adaptation towards stress-tolerant species is buffered by spatial patterning, leading to evolutionary homeostasis over a wide precipitation range. Parameter values are as shown in table 1.

We address this question by applying an adaptive dynamics approach [25–28] to a dryland vegetation model that includes pattern-forming scale-dependent feedback [7,8] and describes evolutionary adaptation to a slowly drying climate involving a species trait shift, making it less fast-growing and more stress-tolerant. A detailed description of the model is provided in §2. Our main finding is illustrated in figure 1. The vegetation patterning shown in figure 1*b* can be highly effective in increasing the resilience to decreasing precipitation, more so than the evolutionary adaptation shown in figure 1*a*, and, as can be expected, yet higher resilience is obtained when the two processes act in concert, as shown in figure 1*c*. Surprisingly, however, that combined response results in *evolutionary homeostasis*, which means that hardly any evolutionary adaptation occurs over a wide precipitation range or, equivalently, during a long time span of dry-climate development. Instead, spatial re-patterning involving patch thinning (or shrinking) and patch dilution (or elimination) takes place, retaining the availability of water to the sparser vegetation and thereby reducing the driving force for further evolutionary adaptation.

**Table 1.** Model parameters, their descriptions, numerical values and units.

parameter	description	value	unit
$\Lambda_{0}$	growth rate at zero biomass	0.032	m^2^ (kg y)^−1^
Γ	water uptake rate	20.0	m^2^ (kg y)^−1^
f	infiltration-contrast parameter (f≪1 means high contrast)	0.01	—
A	maximal value of infiltration rate I	40.0	$y^{-1}$
Q	reference biomass at which I≈A/2 for f≪1	0.06	$kg\;m^{-2}$
$L_{0}$	evaporation rate in bare soil	4.0	y^−1^
R	evaporation reduction due to shading	10.0	$m^{2}\;kg^{-1}$
K	reference biomass for 50% growth attenuation	variable	$kg\;m^{-2}$
$K_{min}$	minimal reference biomass for 50% growth attenuation	0.1	$kg\;m^{-2}$
$K_{max}$	maximal reference biomass for 50% growth attenuation	0.6	$kg\;m^{-2}$
M	mortality rate	variable	$y^{-1}$
$M_{min}$	minimal mortality rate	0.5	$y^{-1}$
$M_{max}$	maximal mortality rate	0.9	$y^{-1}$
Y	relative contribution to infiltration rate	variable	—
$Y_{min}$	minimal relative contribution to infiltration rate	0.5	—
$Y_{max}$	maximal relative contribution to infiltration rate	1.5	—
P	precipitation rate	variable	$mm\;y^{-1}$
χ	trait describing growth-tolerance trade-off	[0,1]	—
$D_{B}$	biomass dispersal rate	1.0	$m^{2}\;y^{-1}$
$D_{W}$	below-ground water diffusion coefficient	$10^{2}$	$m^{2}\;y^{-1}$
$D_{H}$	above-ground water diffusion coefficient	$10^{4}$	$m^{2}\;y^{-1}$
$C_{\chi }$	relative rate of trait evolution	$10^{-3}$	—
$C_{P}$	rate of precipitation decrease	0.011	$mm\;y^{-2}$

This observation raises a second question we examine in this paper: can the evolution towards stress-tolerant and thus less productive species be reduced before the onset of spatial patterning, which largely buffers against further evolution? To address this question, we study factors that affect the onset of spatial patterning, including multi-stability ranges of uniform and patterned states, and effects of spatial heterogeneity in the soil and temporal disturbances in biomass distribution. We begin in the next section by introducing the model we use for studying the combined dynamics of evolutionary adaptation and vegetation patterning.

## 2. Methods

### 2.1. Integrating evolutionary dynamics into vegetation pattern-formation theory

According to the theory of vegetation pattern formation [29], vegetation patchiness in drylands can appear under conditions of water stress even in spatially homogeneous ecosystems. The emergence of patchiness from uniform vegetation is a threshold phenomenon, occurring when the precipitation rate drops below a critical value. It is driven by a positive feedback loop between local vegetation growth and water transport towards the growth location [8]. This is a scale-dependent feedback that facilitates the growth of incidentally denser patches and inhibits the growth in their neighbourhoods, leading to Turing instability [7,30]. Several mechanisms of water transport have been identified [8]: above-ground overland water flow [31,32], below-ground lateral soil-water diffusion [20,33] and water conduction by laterally spread roots [34]. The patterned vegetation state that forms below the Turing-instability threshold persists to lower precipitation rates than the uniform vegetation state, as vegetation patches now benefit from an additional water supply—the water they draw from their bare soil surroundings. As precipitation drops further, patterned vegetation states of longer wavelengths appear and persist at yet lower precipitation rates [35–38].

Besides vegetation patterning, water stress can also induce evolutionary adaptation. Several plant adaptation strategies to water stress have been distinguished, including tolerance, escape and avoidance strategies [39,40]. In the following, we use the term ‘tolerance’ to describe any strategy involving adaptive traits. The higher capacity of plants to tolerate water stress is generally accompanied by lower growth rates [24]. This trade-off can be modelled by introducing a dimensionless trait variable 0≤χ≤1 so that χ=0 represents a plant species investing mostly in growth while χ=1 represents a plant species investing mostly in tolerating water stress [17,41]. As precipitation drops, evolution towards higher χ values is expected. According to adaptive-dynamics theory, this evolution is driven by the selection gradient, that is, the differential increase of plant fitness with changes in χ [28].

We study the interplay between evolutionary adaptation and vegetation patterning using a continuum model [42] that consists of three partial differential equations for the spatial distributions of above-ground biomass B, soil water content W and surface water H and an ordinary differential equation for the trait variable χ, as described below.

### 2.2. Evolutionary vegetation pattern-formation model

We consider dryland ecosystems in flat homogeneous terrains, where bare or sparsely vegetated soil is covered by physical or biogenic soil crusts that reduce the infiltration rate of surface water into the soil [43–45]. Infiltration rates in soil areas covered by dense vegetation remain relatively high, because soil crusts hardly develop there, and plants’ roots increase soil porosity. The infiltration contrast that develops between areas of incidentally denser and sparser vegetation induces above-ground water flow towards the denser vegetation, which makes the denser vegetation yet denser and the water flow faster. This scale-dependent feedback can induce a Turing instability of uniform vegetation resulting in the emergence of spatial patterns [8,31]. We assume for simplicity [46,47] that the other two water transport forms, soil-water diffusion and water conduction by laterally spread roots, are too weak to induce spatial patterning.

A model that captures this scale-dependent feedback in one spatial dimension is [36]


(2.1a) $$\begin{array}{l} \partial_{t}B\;=\;\Lambda (B)WB-MB+D_{B}\partial_{x}^{2}B, \end{array}\;$$

(2.1b) $$\begin{array}{l} \partial_{t}W\;=\;I(B)H-L(B)W-\Gamma WB+D_{W}\partial_{x}^{2}W, \end{array}$$

(2.1c) $$\begin{array}{l} \partial_{t}H\;=\;P-I(B)H+D_{H}\partial_{x}^{2}H, \end{array}$$

where B(x,t) is the areal biomass density of the resident species at location x and time t, and W(x,t) and H(x,t) are the corresponding below-ground and above-ground water densities, respectively, which can all be measured in units of kg m^−2^. The three biomass-dependent functions in equation (2.1) are given by


(2.2a) $$\begin{array}{l} \Lambda (B)\;=\;\Lambda_{0}\left(1-\frac{B}{B+K}\right), \end{array}$$

(2.2b) $$\begin{array}{l} I(B)\;=\;A\frac{YB\;+\;fQ}{YB\;+\;Q}, \end{array}$$

(2.2c) $$\begin{array}{l} L(B)\;=\;\frac{L_{0}}{1\;+\;RB}. \end{array}\;$$

The reader is referred to table 1 for a description of all model parameters, their units and their values. Similar results have been obtained for parameter values other than those used in our figures, including higher precipitation ranges. The scale-dependent feedback is captured by the biomass dependence of the infiltration rate I(B) (equation (2.2b)) for a high infiltration contrast or f≪1 (i.e. infiltration is high in vegetation patches and low in bare soil) and by the term describing surface water transport (last term in equation (2.1c)), modelled for simplicity as a diffusion process [48]. When f≪1 vegetation patches act as sinks for surface water flow from the adjacent bare-soil patches, whereas for f=1 (low infiltration contrast, not considered in this paper), the infiltration rate becomes constant (I=A) and surface water would not flow laterally; rather, it would just infiltrate into the soil. The precipitation rate P represents the mean annual precipitation. Accordingly, B,W and H are interpreted as mean annual densities. We further assume that surface water levels are significant only during short periods of time (hours) after rain episodes, during which evaporation is negligible, and therefore do not include an evaporation term in equation (2.1c). This is unlike soil water content, which remains significant over much longer periods (days, weeks and even months, depending on soil type and depth) during which evaporation is not negligible. The soil-water evaporation rate L(B) (equation (2.2c)) is biomass-dependent because of shading. Finally, we consider late-growth attenuation effects due to self-shading [49] and therefore consider a biomass-dependent growth rate Λ(B) (equation (2.2a)).

Evolutionary adaptation to drier climates is modelled using a trait variable χ that represents a trade-off between a plant’s investment in growth, quantified by the reference biomass K in equation (2.2a) versus investment in tolerance to water stress, quantified by the mortality rate M in equation (2.1a). The effects of the trait-trade-off variable χ are defined through the relations


(2.3a) $$\begin{array}{ll} K(\chi ) & =K_{max}+\chi \left(K_{min}-K_{max}\right), \end{array}\;$$

(2.3b) $$\begin{array}{ll} M(\chi ) & =M_{max}+\chi \left(M_{min}-M_{max}\right). \end{array}$$

Thus, χ=0 represents species investing mostly in growth, attaining the highest reference biomass $K_{max}$ but experiencing the highest mortality rate $M_{max}$, while χ=1 represents species investing mostly in tolerating water stress, experiencing the lowest mortality rate $M_{min}$ but also attaining the lowest reference biomass $K_{min}$. Since bigger plants generally have larger root systems, which increase soil porosity and thus infiltration rates, we further introduce a χ dependence of the relative contribution to the infiltration rate Y


(2.4) $$Y(\chi )=Y_{max}+\chi \left(Y_{min}-Y_{max}\right).$$

As derived analytically in appendix A of [28], the evolutionary dynamics are driven by the spatial average of the local selection gradient ∂G/∂χ according to


(2.5a) $$\begin{array}{l} \frac{d\chi }{dt}\;=\;\frac{C_{\chi }}{\int B^{2}(x)dx}\int B^{2}(x)\frac{\partial G}{\partial \chi }dx, \end{array}\;$$

(2.5b) $$\begin{array}{l} G\;=\;\frac{1}{B}\frac{\partial B}{\partial t}=\Lambda \left(1-\frac{B}{B+K(\chi )}\right)W-M(\chi ), \end{array}$$

where G is the *per capita* growth rate and $C_{\chi }$ is a small parameter that determines the time-scale separation between fast ecological processes and slow evolutionary dynamics. The spatial average of the selection gradient is weighted by the biomass squared [28], giving more weight to locations of denser vegetation. Equations (2.1) and (2.5) describe the coupled dynamics of vegetation patterning and evolutionary adaptation induced by the development of a drier climate. Implicit in this model is the assumption of a single dominant species at any given time, as the model contains a single biomass variable describing this species.

### 2.3. Numerical methods

We solve equations (2.1) and (2.5) numerically on one-dimensional spatial domains with periodic boundary conditions using a spectral method with Runge–Kutta fourth-order time stepping [50]. A uniform vegetation state with small random perturbations is used as the initial condition unless otherwise described. Temporal biomass disturbances of a uniform vegetation state are introduced by time-periodic local biomass-removal events, with each event removing 15% of the total area in two locations drawn from a uniform distribution. Spatial soil heterogeneities are introduced through random spatial distributions of the infiltration-contrast parameter f with values of f drawn from a normal distribution with a mean of 0.01 and smoothed by a Gaussian filter. The standard deviation (s.d.) and the auto-correlation function (ACF) of these distributions are calculated, with ACF normalized to a maximum value of 1. The auto-correlation length (ACL) is estimated as the distance at which the ACF first falls below the threshold value 1/e [51].

## 3. Results

### 3.1. Evolutionary dynamics of a non-pattern-forming system

It is instructive to first consider the case of biotic and abiotic conditions that rule out the formation of vegetation patterns along the rainfall gradient. In the model given by equation (2.1), this applies when the infiltration rate I hardly depends on biomass, that is, when the infiltration-contrast parameter f is close to 1. This is often the case in sandy soils where the infiltration rate in bare soil can be as high as in vegetation patches.

**Figure 2.**
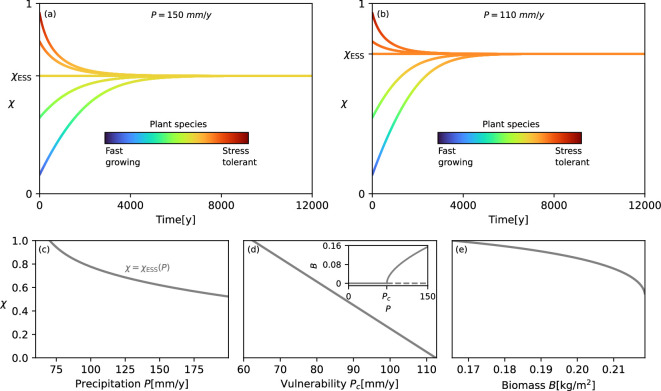
Evolutionary adaptation of spatially uniform vegetation to decreasing precipitation. (*a*) Adaptive dynamics trajectories for a precipitation rate of P = 150 mm y^−1^, showing convergence to a unique evolutionarily stable strategy, $\chi_{ESS}=0.62$. (*b*) Analogous adaptive dynamics for a lower precipitation rate P=110 mm y^−1^ resulting in a species that better tolerates water stress, $\chi_{ESS}=0.735$. The change of colours along the trajectories shows the adaptive dynamics according to the colour bars. (*c*) Evolutionary adaptation from fast-growing to stress-tolerant species as precipitation decreases. (*d*) The decreasing vulnerability associated with evolutionary adaptation, quantified by the precipitation threshold $P_{c}$, at which the uniform vegetation state ceases to exist; see the bifurcation diagram in the inset. (*e*) Decreasing biomass production associated with evolutionary adaptation for P=200 mm y^−1^. Parameter values are as shown in table 1.

Solving equations (2.1) and (2.5) for a given precipitation rate, we find that the evolutionary dynamics result in trajectories χ(t) that converge to a unique trait value, representing an evolutionarily stable strategy $\chi_{ESS}$ [52], irrespective of the initial trait value. Figure 2*a*,*b* shows such trajectories for two distinct precipitation rates, P=150 mm y^−1^ and P=110 mm y^−1^. The change of colours along the trajectories shows the course of evolutionary adaptation. The dynamics for the higher precipitation rate converge to a higher χ value ($\chi_{ESS}=0.62$), representing faster-growing, less-tolerant species, than those for the lower precipitation rate ($\chi_{ESS}=0.735$). As the shown bundles of evolutionary trajectories indicate, evolutionary dynamics starting from different initial trait values culminate in the same evolutionarily stable strategy, both in figure 2*a* and in figure 2*b*.

The evolutionary adaptation from fast-growing (low χ) to stress-tolerant (high χ) species along the rainfall gradient is shown in figure 2*c*. This adaptation decreases the modelled ecosystem’s vulnerability to droughts (increases its resilience), as figure 2*d* shows. The vulnerability is defined here as the precipitation threshold $P_{C}$ at which the uniform vegetation state ceases to exist (inset in figure 2*d*); the higher the $P_{C}$, the sooner the transition to bare soil occurs as precipitation drops, and thus the higher the vulnerability. Importantly, the evolutionary adaptation from fast-growing to stress-tolerant species is accompanied by a reduction in biomass production, as figure 2*e* shows. These results underlie the first question we have posed in §1, namely, whether patterning can inhibit undesired evolutionary adaptation towards less-productive species without compromising ecosystem resilience. We address this question in the next two subsections.

### 3.2. Emergence of evolutionarily stable patterns along the rainfall gradient

**Figure 3.**
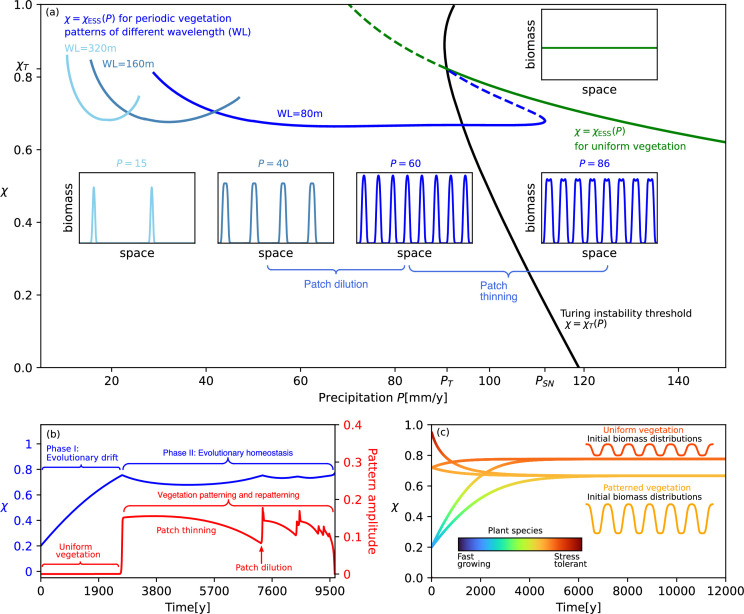
Steady-state model solutions representing evolutionarily stable strategies along the rainfall gradient and the dynamics they imply. (*a*) Bifurcation diagram showing a steady-state uniform vegetation solution (green line) and several steady-state periodic vegetation solutions of increasing wavelength (WL) as precipitation decreases (with blue lines of lighter-blue colours denoting longer-wavelength patterns), along with examples of their spatial biomass distributions (insets). Solid (dashed) lines depict stable (unstable) solutions (with the unstable parts of the periodic solutions in the low P range not shown). The precipitation rate $P_{T}$ denotes the Turing-instability threshold of the uniform vegetation solution that represents an evolutionarily stable strategy $\chi_{T}=\chi_{ESS}(P_{T})$. The precipitation rate $P_{SN}$ denotes the saddle-node bifurcation threshold at which the short-wavelength solution (dark-blue line) ceases to exist. (*b*) Evolutionary adaptation (blue line) and spatial patterning (red line), in response to precipitation decreasing at a rate $C_{P}=0.011$ (mm y^−2^). The dynamics involve two phases: evolutionary adaptation when the vegetation is still uniform (phase I), followed by evolutionary homeostasis induced by spatial patterning and re-patterning (phase II). The latter includes patch thinning along solution branches and patch dilution at transitions to longer wavelength solutions. (*c*) Selection of an evolutionarily stable strategy according to initial spatial biomass distributions in a bistability range of uniform and patterned states. Perturbations of the uniform vegetation state decay when their amplitude is sufficiently small, resulting in a species with a relatively high $\chi_{ESS}$ value, irrespective of the initial χ value. In contrast, perturbations of sufficiently high amplitude grow and converge to periodic patterns, which trigger adaptive dynamics towards a lower $\chi_{ESS}$ value because of lower water stress. The change of colours along the trajectories shows the evolutionary dynamics according to the colour bars. Parameter values are as shown in table 1.

Vegetation patterning acts to relax local water stress by allowing plants to utilize the water from adjacent bare-soil patches. When such patterning occurs on ecological time scales that are much shorter than the evolutionary time scales, we expect it to interfere strongly with evolutionary adaptation by weakening its driving force. To study the effect of vegetation patterning on adaptation to a drier climate, it is instructive to consider how evolutionarily stable uniform and patterned solutions $\chi_{ESS}$ change with the precipitation rate P. Figure 3*a* shows a bifurcation diagram of such solutions in the plane spanned by χ and P, comprising a stationary uniform solution branch (green line) and patterned solution branches of increasing wavelengths (blue lines). Superimposed on this diagram is the Turing-instability threshold $\chi_{T}(P)$ (black line) at which a uniform vegetation state of a species with trait value χ loses stability to periodic patterns. The solution of $\chi_{ESS}(P)=\chi_{T}(P)$, i.e. the intersection of the black line with the green line, determines the Turing-instability threshold, $P=P_{T}$, of an evolutionarily stable uniform vegetation state together with the corresponding threshold trait value $\chi_{T}=\chi_{ESS}(P_{T})$. The dashed segment of the green line represents a uniform vegetation state that is unstable to non-uniform spatial perturbations but is still evolutionarily stable in the absence of such perturbations.

A comparison of the uniform solution branch (green line) with the patterned solution branch that bifurcates from it at the Turing-instability threshold $P=P_{T}$ (dark-blue line) reveals a striking difference: while the former shows a steep ascent towards lower precipitation values, representing evolutionary adaptation towards stress-tolerant species, the latter is horizontal over a wide precipitation range, showing virtually no evolutionary change. This is caused by differential plant mortality, which makes vegetation patches thinner (compare insets at P=86 and 60 mm y^−1^) and weakens the competition for water. The reduced water stress due to weaker competition for water counterbalances the increased stress due to lower precipitation and thus keeps $\chi_{ESS}(P)$ largely unchanged.

Patch thinning is effective down to a certain precipitation rate (approx. 60 mm y^−1^), below which evolutionary adaptation towards stress-tolerant species begins. However, at this precipitation range, another stable periodic solution with fewer vegetation patches, or longer wavelength, exists, as the longer-wavelength solution branch in figure 3*a* (intermediate-blue line) and the corresponding inset at P=40 mm y^−1^ show. Just like patch thinning, patch dilution occurring through the elimination of some patches acts to counterbalance the increased water stress resulting from drier conditions and, thereby, keeps $\chi_{ESS}$ small. As precipitation decreases further, a yet longer-wavelength solution branch (light-blue line) appears, playing a similar role in weakening the driving force of evolutionary adaptation and continuing to keep $\chi_{ESS}$ small.

### 3.3. Evolutionary homeostasis

The bifurcation diagram in figure 3*a* provides insights about possible vegetation responses to decreasing precipitation. When precipitation varies on a time scale much longer than that of evolutionary dynamics, the latter tracks the stable parts of the solution branches in the diagram. In this case, when the Turing-instability threshold at $P=P_{T}$ is traversed as the precipitation rate P is decreased (formally speaking, at an infinitely slow rate), the evolutionarily stable uniform vegetation state becomes unstable to non-uniform spatial perturbations, and a sharp transition to a periodic pattern determined by the corresponding threshold trait value $\chi_{T}=\chi_{ESS}(P_{T})$ occurs. This is followed by evolutionary adaptation to the patterned state, which is slow compared with the vegetation patterning and fast compared with the precipitation drop. The transition to a patterned state involves a decrease in χ, as the water-stress relaxation associated with patterning favours faster-growing species. From there on, virtually no evolutionary adaptation takes place for a wide precipitation range; the system follows the periodic solution branch at a largely constant $\chi_{ESS}$ value, as the dark-blue line in figure 3*a* shows. We refer to this phase of the dynamics as *evolutionary homeostasis*.

In practice, a separation of time scales between precipitation decrease and evolutionary dynamics may not exist, but the distinction between two phases—evolutionary adaptation towards stress-tolerant species and evolutionary homeostasis—still applies. This is evidenced by the blue line in figure 3*b*, which shows the evolutionary trajectory χ=χ(t), in response to precipitation that decreases on a time scale comparable to that of evolutionary adaptation. The initial state is uniform vegetation of a fast-growing species (χ=0.2), and while that species evolves towards an evolutionarily stable strategy representing a more stress-tolerant species (higher χ), the precipitation decreases as well. As a result, the Turing-instability threshold (black line in figure 3*a*) is hit before the evolutionarily stable uniform state is reached, that is, at $P>P_{T}$ and $\chi <\chi_{T}$, and a fast transition to a patterned state occurs, as indicated by the sharp increase in the pattern amplitude depicted by the red line in figure 3*b*. That transition brings to an end the phase of steady evolutionary adaptation to stress-tolerant species, that is, phase I of the response dynamics. The subsequent evolutionary adaptations oscillate back and forth within a narrow χ range, reflecting the approach to the lower $\chi_{ESS}$ value of the patterned state, and the processes of patch thinning and patch dilution. This is phase II of the response dynamics, during which the high spatial plasticity of vegetation patterns, associated with patch thinning and patch dilution, leads to evolutionary homeostasis. Phase II comes to an end in a collapse to bare soil, as the pattern’s wavelength becomes large compared with the range of above-ground water flow, and longer-wavelength patterns are ineffective in relaxing the water stress.

### 3.4. Pattern-induced evolutionary bistability

A common aspect of all spatial vegetation models [31,33,35,53–55] is the existence of a precipitation range over which the uniform vegetation state and patterned vegetation states are both stable. This occurs when the precipitation rate is sufficiently high to support uniform vegetation, and the relevant scale-dependent feedback is sufficiently strong to support patterned vegetation. In the current model, the bistability of uniform and patterned vegetation exists over the range $P_{T}<P<P_{SN}$ (figure 3*a*). In this range, weak spatially heterogeneous perturbations of uniform vegetation fade out, while strong spatially heterogeneous perturbations can induce a transition to patterned vegetation. Since the driving force of evolutionary adaptation is weaker in patterned vegetation than in uniform vegetation, as bare-soil areas provide additional water to adjacent vegetation patches and thereby reduce their water stress, we may expect species in patterned vegetation to evolve towards lower χ values than those in uniform vegetation. The bistability of uniform and patterned vegetation thus implies an evolutionary bistability as shown in figure 3*c*. Low-amplitude spatially periodic perturbations of a uniform vegetation state of a species with trait value χ fade out and induce slow adaptive dynamics towards a less-productive, more stress-tolerant species, while high-amplitude spatially heterogeneous perturbations grow and converge to a patterned state, thereby inducing adaptive dynamics towards more productive, less stress-tolerant species. Figure 3*c* also demonstrates that the resulting evolutionarily stable strategy, $\chi_{ESS}$, is independent of the initial trait value; the same initial trait value χ can evolve to different $\chi_{ESS}$ values, and different initial trait values χ can evolve to the same $\chi_{ESS}$ value, depending on the initial spatial biomass distribution. This independence applies whenever vegetation patterning is sufficiently faster than evolutionary adaptation.

### 3.5. Productivity decline

**Figure 4.**
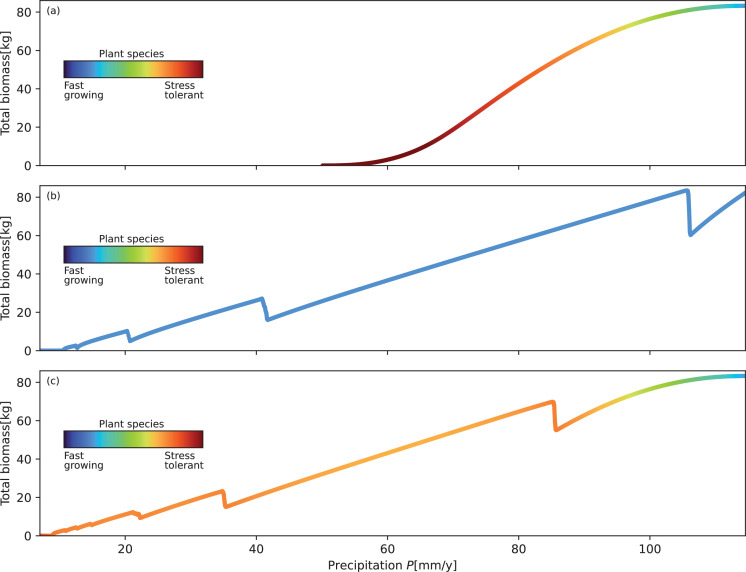
Total biomass decline with decreasing precipitation for the three scenarios in figure 1. (*a*) Evolutionary adaptation in the absence of vegetation patterning. (*b*) Vegetation patterning in the absence of evolutionary adaptation. (*c*) Combination of vegetation patterning and evolutionary adaptation. The change of colours along the trajectories shows the adaptive dynamics according to the colour bars. The biomass jumps at high precipitation rates indicate transitions from uniform to patterned vegetation. The subsequent jumps at lower precipitation rates indicate transitions to longer-wavelength patterns. Domain size is 1000 m. Parameter values are as shown in table 1.

While $\chi_{ESS}$ hardly changes as precipitation is decreased over the wide range of spatial patterns, productivity, as measured by the total biomass, is strongly reduced. Figure 4 shows the total biomass change for the three scenarios in figure 1, with the precipitation rate P decreasing at a slow constant rate $C_{P}$ from a high value that enables stable uniform vegetation. The strongest total biomass decline occurs in the scenario of evolutionary adaptation alone (figure 4*a*). This biomass decline is monotonic, starting shallowly but then becoming steeper, eventually leading to the complete extinction of the most stress-tolerant species at an intermediate precipitation rate (P≈50 mm y^−1^). Spatial patterning drastically changes this trend; the biomass decline is not monotonic, as spatial patterning and pattern transitions to longer-wavelength patterns induce fast biomass upshifts, and extinction occurs at much lower precipitation values (figure 4*b*,*c*). However, without evolutionary adaptation the initial biomass decline, before spatial patterning begins, is much steeper compared with the case of evolutionary adaptation (compare the high precipitation ranges in figure 4*a*,*b*). Combined spatial patterning and evolutionary adaptation result in the most desirable response dynamics, involving a shallow initial biomass decline, complete extinction at very low precipitation and homeostasis over a wide precipitation range, as shown by the trajectory’s nearly uniform colour below P≈90 mm y^−1^ (figure 4*c*).

### 3.6. Effects of temporal disturbances and spatial heterogeneities

The second question we have posed in §1 is whether evolution towards stress-tolerant and thus less-productive species can be slowed down before the onset of spatial patterning, which largely buffers against further evolution (figure 4*c*). We address this question by introducing temporal biomass disturbances and spatial soil heterogeneities as described below.

**Figure 5.**
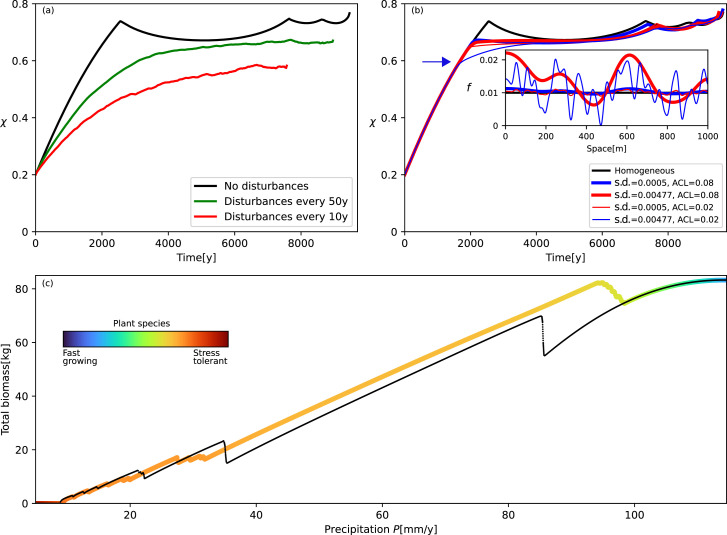
Effects of temporal disturbances and spatial heterogeneities. (*a*) Time-periodic biomass-removal disturbances moderate the evolutionary adaptation from fast-growing to stress-tolerant species. Shown are trajectories χ(t) as precipitation linearly drops, in the case of no disturbances (black line), and for disturbances that are random in terms of their spatial size and location, and periodic in terms of their temporal occurrence; every 50 years (green line) or 10 years (red line). More frequent disturbances result in faster-growing species, but also in earlier collapse to bare soil as the termination points of the trajectories indicate. (*b*) Random spatial heterogeneity, introduced through the infiltration-contrast parameter f, stops the evolutionary adaptation from fast-growing to stress-tolerant species, as precipitation linearly drops, by expediting spatial patterning. Shown are evolutionary trajectories for a spatially homogeneous system (black line) and for heterogeneous systems characterized by four different combinations of ACL and s.d., as indicated in the legend. The inset shows realizations of the random spatial heterogeneity for these four combinations. Shorter ACL and larger s.d. (thin blue line) are more effective in delaying the evolutionary adaptation to stress-tolerant species by inducing spatial patterning earliest, as highlighted by the blue arrow. (*c*) Total biomass decline with decreasing precipitation for the heterogeneous system represented by the thin blue line in (*b*). The biomass decline (thick line with changing colours) is altogether weaker and occurs later than for the corresponding homogeneous ecosystem shown in figure 4*c* (thin black line). Domain size is 1000 m. Parameters are as shown in table 1.

Temporal biomass disturbances of a uniform vegetation state are introduced as described in §2. The effect of biomass disturbances in moderating evolutionary adaptation to less productive stress-tolerant species is expected to be effective in the bistability precipitation range of uniform and patterned vegetation (figure 3*a*), where disturbances may induce a state transition from uniform vegetation to periodic patterns before the Turing instability is reached. Figure 5*a* shows evolutionary adaptation to decreasing precipitation for an undisturbed, spatially homogeneous ecosystem as well as for temporally disturbed but otherwise spatially homogeneous ecosystems in which disturbances occur either every 50 years or every 10 years, starting with a fast-growing species (χ=0.2). The higher the disturbance frequency, the more moderate the evolutionary adaptation towards stress-tolerant species, but also the sooner the collapse to bare soil. This happens because the considered temporal disturbances enable earlier transitions to patterned vegetation and thereby reduce the driving force of evolutionary adaptation towards stress-tolerant species.

Spatial soil heterogeneities are introduced as described in §2, through random distributions of the infiltration-contrast parameter f, for four different combinations of s.d. and ACL. The effects of spatial heterogeneity are shown in figure 5*b*. Shorter ACLs (distinguished by line thickness) and larger s.d. (distinguished by line colour) are both more effective in inhibiting evolutionary adaptation towards stress-tolerant species by inducing an early transition to patterned vegetation. The shorter of the two shown ACLs is comparable to the typical patch size of the Turing pattern and, therefore, is more effective in inducing the transition to patterns. The larger of the two shown s.d. increases the probability of leaving the basin of attraction of the uniform vegetation state and converging to a patterned vegetation state. Among the four shown cases, the combination of shorter ACL and larger s.d. (thin blue line in figure 5*b*) results in the earliest onset of spatial patterning occurring at the smallest χ value (blue arrow in figure 5*b*). Unlike the temporal biomass disturbances (figure 5*a*), earlier transitions to patterned vegetation, caused by spatial soil heterogeneities, are not compromised by an early collapse to bare soil, which can be considered advantageous from the perspective of vegetation management. As the transition to patterned vegetation caused by spatial soil heterogeneities occurs earlier in the evolutionary adaptation towards less-productive, stress-tolerant species, compared with homogeneous ecosystems, the resultant biomass decline is mitigated, as figure 5*c* shows, which can again be considered advantageous from the perspective of vegetation management.

## 4. Discussion

In this work, we have integrated the theories of adaptive dynamics and vegetation pattern formation to study vegetation response to a slowly developing drier climate, focusing on the effects of vegetation patterning on the evolutionary adaptation of fast-growing species towards stress-tolerant ones. The following two distinct response phases have been identified as drier conditions develop: (i) evolutionary adaptation of spatially uniform vegetation towards less-productive, more stress-tolerant plant species; (ii) evolutionary homeostasis during which spatial patterning and plasticity retard further evolutionary adaptation and yet increase vegetation resilience to water stress. We have also demonstrated how temporal disturbances and spatial heterogeneities induce vegetation patterning at an earlier stage of the evolutionary adaptation towards stress-tolerant species, thereby mitigating the productivity loss that otherwise occurs while the vegetation remains uniform.

Plants actively suppress growth under stressed conditions as an adaptive strategy to improve their tolerance to environmental pressure. This is often achieved by a stress-signalling network that inhibits cellular anabolic activities and plant growth while activating mechanisms to prevent and repair cellular damage [24]. The resultant inherent trade-off between growth and stress tolerance bears negatively on crop productivity. Attempts at increasing crop productivity by means of genetic intervention have so far been of limited success [56,57]. Our results point towards a possible alternative direction for evading the negative implications of this trade-off—self-organization in spatial patterns [58]. The high spatial plasticity of such patterns, enabled by patch thinning, patch dilution and morphology changes (see our discussion of two-dimensional responses below), inhibits evolutionary adaptation towards less-productive crops without compromising their resilience to droughts.

The capability to self-organize in spatial patterns depends on the existence and strength of scale-dependent feedback [7,8]. The feedback included in equation (2.1) is associated with above-ground water flow towards vegetation patches and has been chosen here because of its robustness in natural dryland ecosystems and the relative ease of its implementation in the adaptive-dynamics framework. It especially applies to drylands where bare soil is covered by physical or biogenic soil crusts that reduce the infiltration of surface water into the soil and thereby generate above-ground water flow towards vegetation patches. The results reported here are not expected to depend on the particular scale-dependent feedback that is responsible for spatial patterning. Other scale-dependent feedbacks, associated with soil-water diffusion [33] and with water conduction by laterally spread roots [34], are expected to yield similar results. This is because they all share the crucial property of relaxing water stress by spatial patterning and maintaining water availability in drier conditions by patch thinning, patch dilution and morphology changes.

For simplicity, we have confined ourselves to one spatial dimension. The results are, therefore, applicable to one-dimensional stripe patterns, such as vegetation bands on gentle slopes [59]. Evolutionary homeostasis is also expected in two-dimensional patterns. In that case, in addition to patch thinning and patch dilution, morphological changes occur [8]. Hexagonal gap patterns, emerging at the Turing-instability threshold, transform into stripe patterns as precipitation decreases, and stripe patterns transform into hexagonal spot patterns as precipitation decreases further. These morphological changes increase the bare-soil areas that surround vegetation patches and, consequently, the amount of water that plants can draw from their surroundings. These processes compensate for the decrease in precipitation and thereby maintain local water availability. They, therefore, are expected to cause evolutionary homeostasis, just as reported here.

We are not aware of empirical data that would be available to test the prediction of evolutionary homeostasis induced by vegetation patterning and the spatial plasticity it entails. Yet, candidate systems for testing this prediction can be proposed. Quite often, a dominant pattern-forming species persists over a wide precipitation range. Examples include *Larrea tridentata* (‘creosote bush’) in North America [60], *Acacia aneura* (‘mulga’) in Western Australia [16,61] and *Combretum micranthum* in Western Africa [62]. We suggest that long-term remote sensing of such systems and image analysis of the resultant data in the wake of biomass changes and indications of spatial plasticity under climate drying trends may provide empirical evidence for the theoretical findings we have reported.

We have focused on water stress as the driving force of evolutionary adaptation and vegetation patterning, but evolutionary homeostasis can be expected to occur also for other forms of resource stress that drive both adaptation and patterning. Several candidate systems can be imagined [7], including bog patterns in wetlands for which nutrient stress has been proposed to cause pattern-forming scale-dependent feedback [63] and salt-marsh ring patterns [64].

It is interesting to place our findings in a broader context of fast, stress-induced ecological processes that can inhibit evolutionary adaptation to the same stress. We specifically refer to phenotypic plasticity, through which a shift in the distribution of phenotypes towards higher fitness can shield the underlying genotypes from natural selection [65,66]. An early example is the thermoregulatory behaviour of lizards, which can inhibit selection for an evolutionary adaptation in their thermal physiology with altitude [67]. This example has later been generalized to other regulatory behaviours, coining the term ‘Bogert effect’ [65,68]. Confronting the Bogert effect is the plasticity-first hypothesis, according to which phenotypic plasticity in a given trait dimension facilitates evolutionary adaptation by allowing a population to persist under environmental change long enough for genetic change to occur, provided genetic variability exists in the considered trait dimension [69–71]. Our results suggest that spatial patterning of plant populations can inhibit their evolutionary adaptation, just as phenotypic changes do according to the Bogert effect. This capacity of vegetation patterning to inhibit evolutionary adaptation may be especially effective because of the high spatial plasticity of vegetation patterns along temporal and spatial environmental gradients.

Finally, a remark about time scales. Three time scales, or rates, are significant for the responses of plant communities to drying climates: the rate at which the mean annual precipitation drops, the rate of evolutionary adaptation and the rate of vegetation patterning. We have considered here the case of fast ecological processes (vegetation-state transitions) relative to the rates of precipitation drop, which largely dictates the rate of evolutionary adaptation, and assumed that the latter two rates are of the same order of magnitude, $d\chi /dt∼P^{-1}|dP/dt|\;=C_{P}/P$. A different rate regime corresponds to eco-evolutionary dynamics, with ecological processes and evolutionary adaptations proceeding on similar time scales, enabling complex feedback dynamics [72–74]. This alternative rate regime may apply to the slow re-patterning of woody (tree) vegetation involving patch dilution (transitions to longer-wavelength patterns) but is less likely to apply to processes of patch thinning and herbaceous vegetation [33]. Other alternative rate regimes of potential interest involve fast precipitation drops, which may result in rate-dependent tipping [75] or fast evolutionary adaptations, through which rate-dependent tipping may be evaded [76]. The effect of vegetation pattern formation on evolutionary adaptation in these alternative rate regimes is an open problem that calls for further studies.

## Data Availability

The code for numerically integrating the model equations for reproducing our results or producing additional numerical data is available at [77].
